# Severe acute pancreatitis induced by primary hyperparathyroidism in pregnancy: a case report and literature review

**DOI:** 10.1093/jscr/rjaf161

**Published:** 2025-03-28

**Authors:** Xusheng Liu, Bo Li, Jianxiao Liu, Shengbing Zhao

**Affiliations:** The First Clinical Medical College of Lanzhou University, No. 11, Donggang West Road, Chengguan District, Lanzhou 730000, China; General Surgery Department, The First Hospital of Lanzhou University, No. 11, Donggang West Road, Chengguan District, Lanzhou 730000, China; The First Clinical Medical College of Lanzhou University, No. 11, Donggang West Road, Chengguan District, Lanzhou 730000, China; General Surgery Department, The First Hospital of Lanzhou University, No. 11, Donggang West Road, Chengguan District, Lanzhou 730000, China

**Keywords:** pregnancy, severe acute pancreatitis, hypercalcaemia, primary hyperparathyroidism, parathyroid adenoma

## Abstract

We report a rare case of severe acute pancreatitis with hypercalcaemia induced by primary hyperparathyroidism (PHPT) in a 27-year-old female patient at 28 weeks of gestation during pregnancy. The patient initially presented with onset epigastric pain with nausea and vomiting and was diagnosed with severe acute pancreatitis, hypercalcaemia, and PHPT. After conservative management, the patient underwent a caesarean section and successfully delivered a baby boy, who suffered from hypocalcaemic convulsions in the postnatal period. The patient’s blood calcium level was still elevated and subsequently diagnosed as a left parathyroid adenoma by single photon emission computed tomography, and her blood calcium returned to normal after parathyroidectomy. PHPT in pregnancy is often difficult to diagnose early because of the non-specific clinical manifestations. Early recognition and timely surgical treatment are crucial for the safety of the mother and baby. In patients with PHPT with moderate to severe hypercalcaemia, parathyroidectomy is recommended in mid-pregnancy.

## Introduction

Primary hyperparathyroidism (PHPT) is a disorder in which calcium metabolism is affected by excessive secretion of parathyroid hormone (PTH) by the parathyroid glands and is the most common cause of hypercalcaemia, which is mainly caused by a single parathyroid adenoma (85%) [1]. PHPT is common in postmenopausal women and rarer in pregnancy, and PHPT in pregnancy complicated by hypercalcaemic severe acute pancreatitis is extremely rare [2]. The symptoms of PHPT in pregnancy are similar to those of normal pregnancy, and physiological changes in calcium homeostasis during pregnancy may mask the biochemical manifestations of PHPT, making early recognition and diagnosis of PHPT in pregnancy extremely challenging. PHPT is extremely hazardous to both the mother and the foetus, and we report a case of severe hypercalcaemia and severe acute pancreatitis associated with PHPT caused by a parathyroid adenoma in the late stages of pregnancy to improve clinicians’ understanding and treatment of this disease.

## Case presentation

**Figure 1:**
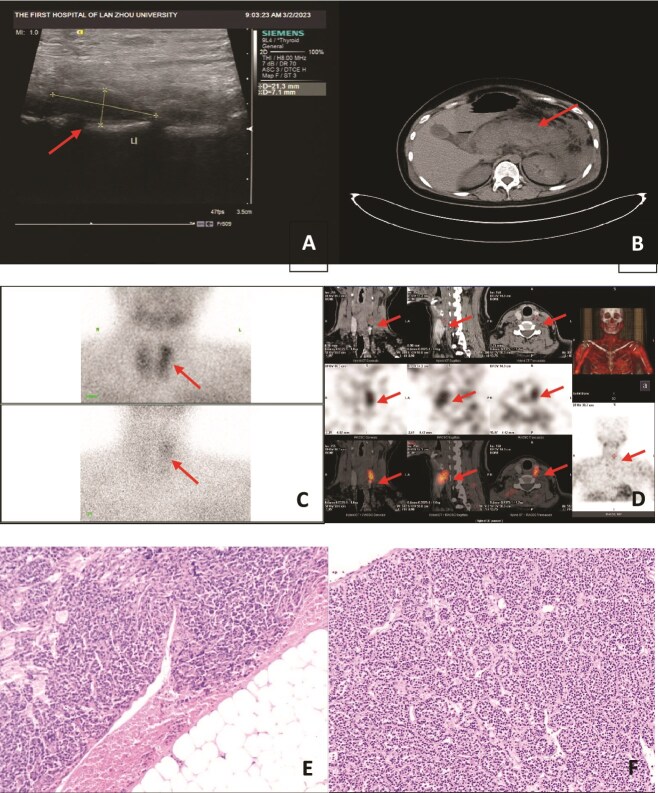
(A) Hypoechoic area posterior to the upper pole of the left lobe of the thyroid gland; (B) acute pancreatitis with necrotic changes, with acute necrotic material accumulation around the pancreas; (C, D) Imaging after 30 min of intravenous injection of 99mTc-MIBI: the thyroid gland was still clear, and a dense area of abnormal tracer distribution was seen above the left lobe of the thyroid gland; imaging after a delay of 2 hr showed that the tracer distribution in the glands of both lobes of the thyroid gland was markedly reduced, and a dense area of abnormal tracer distribution was still seen above the left lobe of the thyroid gland; (E, F) morphological and immunohistochemical results support parathyroid adenoma (HE staining, 10 × 10).

The patient was a 27-year-old woman. She was admitted to the hospital for ‘sudden epigastric pain with nausea and vomiting for 1 day at 28 weeks of pregnancy’. She was physically fit, with no history of long-term use of calcium and vitamins; no history of alcohol consumption or greasy food; and no family history of specific diseases. The patient had regular menstruation, with her last menstrual period being 6 months ago, G2P1, and no history of abnormal vaginal bleeding. Admission examination: body temperature 37.8°C, pulse 93 times/min, respiration 24 times/min, blood pressure 146/88 mmHg (1 mmHg = 0.133 kPa), body mass index 30 kg/m^2^. Uterus size in line with the gestational week, left upper abdomen and mid-abdomen abdominal muscle tense, obvious pressure pain, rebound pain. Auxiliary tests: routine blood tests: red blood cell count 3.17 × 10^12^/L, white blood cell count 16.63 × 10^9^/L, neutrophil percentage 90.4%. Hepatopancreatic function: ALP 220.9 U/L, AMY 2070 U/L, LPS 299 U/L. Laboratory blood tests: Ca 3.69 mmol/L, PTH 236.0 pg/ml; C-reactive protein 20.58 mg/L, IL-6208.0 pg/ml, calcitonin 23.600 ng/ml. Thyroid ultrasound showed: (i) hypoechoic area posterior to the upper pole of the left lobe of the thyroid gland (parathyroid gland occupation was considered) (Fig. 1A). Abdominal ultrasound showed: (i) pancreas showed partial volume increase; (ii) ascites; and (iii) no gallbladder stones. Abdominal CT showed: (i) acute pancreatitis with necrotic changes and accumulation of acute necrotic material around the pancreas (Fig. 1B); (ii) abdominopelvic fluid; and (iii) uterus in pregnancy.

The working diagnosis was severe acute pancreatitis secondary to primary hypercalcaemia. The treatment plan was: (i) for hypercalcaemia: about 2000 ml of fluid supplementation per day, intermittent application of diuretics and (ii) for severe acute pancreatitis: gastrointestinal decompression, laxative, diuretic, fluid supplementation, anti-inflammatory, acid suppression, enzyme suppression, peritoneal fluid, and peripancreatic fluid drainage. Two days later, the patient had shortness of breath, and lethargy, accompanied by hypo-proteinaemia, and was transferred to ICU for supportive therapy. After repeated communication with the patient and her family, the patient and her family gave up foetal preservation, and the patient underwent caesarean section under lumbar anaesthesia, during which the amniotic fluid was seen to be contaminated to the I degree, with a large amount of about 2000 ml, and the delivery of a live male baby was assisted with underdevelopment, and the baby’s blood calcium was 3.39 mmol/L on the day of delivery, and then dropped to 1.63 mmol/L 2 days later, accompanied by hypocalcaemic convulsions, and the preterm baby was sent to the NICU for further treatment. The patient’s condition stabilized after symptomatic supportive treatment in the ICU, and then she was transferred to our department, and eventually, the patient and the neonate were discharged from the hospital with improvement. After the patient was discharged from the hospital, the blood calcium continued to rise, accompanied by weakness of the limbs, so the patient was discharged from the hospital for 1 month and re-admitted to our hospital, and the SPECT parathyroid gland showed: abnormal tracer concentration area above the left lobe of the thyroid gland (Fig. 1C and D), combined with the tomographic fusion images, and considered: the left upper parathyroid gland hyperfunction (mild). Under general anaesthesia, left parathyroid adenoma resection was performed. Rapid intraoperative PTH (ioPTH) measurement: 37.9 pg/ml, resection was successful, and postoperative histopathology supported: parathyroid adenoma (Fig. 1E and F). Postoperative PTH: 49.7 pg/ml, Ca 2.17 mmol/L. She was discharged on the third postoperative day.

## Discussion

The clinical manifestations of patients with PHPT during pregnancy are related to PHPT on the one hand, and their severity can progress from mild, non-specific discomfort manifestations to pathological changes caused by an imbalance of calcium homeostasis in various organs, including skeletal system involvement (e.g. bone pain, osteoporosis, etc.), urological symptoms (e.g. polydipsia, renal stones, etc.), and gastrointestinal symptoms associated with hypercalcaemia (e.g. nausea, vomiting, pancreatitis, etc.); On the other hand, it is closely associated with maternal and foetal/neonatal complications during pregnancy, such as severe vomiting of pregnancy, pre-eclampsia, amniotic fluid excess, neonatal hypocalcaemic convulsions, and even miscarriage [3, 4]. Our patient developed vomiting, abdominal pain, and a rare complication of PHPT, hypercalcaemic pancreatitis, in late pregnancy. The development of pancreatitis may be related to the following mechanisms: (i) hypercalcaemia increases the calcium concentration in the pancreatic fluid and promotes the secretion of trypsinogen; (ii) it causes pancreatic stones to obstruct the pancreatic ducts; (iii) it accelerates the conversion of trypsinogen into trypsin, which attacks the pancreatic parenchyma and pancreatic ducts; and (iv) elevated levels of PTH cause pancreatic microthrombi, which can directly damage the pancreas [5]. Hypocalcemic tetany occurs in the postnatal period due to the active transport of calcium from the mother to the foetus during gestation leading to secondary hypercalcaemia in the foetus, this process is interrupted after delivery and hypocalcemic tetany occur as a result of the inability of the mother’s PTH to pass through the placental membranes and the prolonged suppression of foetal parathyroid function, which prevents the newborn from mobilizing endogenous calcium reserves [6].

PHPT during pregnancy leads to increased maternal and foetal complications and mortality and should be diagnosed early and clearly for optimal management. Pregnant women with risk factors for hypercalcaemia such as previous kidney stones should be screened for PHPT during pregnancy. The need for calcium increases dramatically during pregnancy due to foetal growth and development, and calcium is actively transported to the foetus through the placenta. In addition, the increase in maternal blood volume during pregnancy results in a relative decrease in blood calcium concentration; the increase in glomerular filtration rate increases urinary calcium excretion, and ultimately the mother is in a low calcium state during pregnancy. Therefore, pregnant women with high blood calcium levels should be alert to other causes of hypercalcaemia such as PHPT [1].

The presence of unexplained nausea, vomiting, and abdominal pain during pregnancy should alert us to the possibility of acute pancreatitis. The common causes of acute pancreatitis include biliary, hyperlipidaemic, alcoholic, and idiopathic, etc. Biliary pancreatitis is common in our country, and the most common biliary pancreatitis was excluded from our patient’s examination. It is well known that blood calcium is low during the acute attack of pancreatitis, so patients with normal or high blood calcium in vomiting with pancreatitis need to pay attention to this as a clue of concomitant PHPT, in order to reduce the underdiagnosis of PHPT. Meanwhile, patients with PHPT should be closely observed for pancreatic function, and attention should be paid to the treatment of high blood calcium to avoid inducing pancreatitis [7]. Therefore, combining these studies on the interrelationship between pregnancy, PHPT, and pancreatitis, we concluded that this patient had an occult parathyroid adenoma, and that pregnancy-induced bleeding from the parathyroid adenoma and exacerbated the hypercalcaemia. It also further aggravated the risk of acute pancreatitis due to the enlargement of the uterus in late pregnancy increasing pancreatic duct pressure and pancreatic secretion [8]. The patient was eventually diagnosed with PHPT based on clinical presentation and examination.

After the qualitative diagnosis of PHPT is completed, further localisation diagnosis is required. The most commonly used screening tests for the localisation of PHPT are neck ultrasound and 99mTc-MIBI dual-phase imaging. To avoid radiation exposure to the mother and foetus, CT and positron emission tomography should be avoided during pregnancy. Neck ultrasound is currently the primary safe method for localisation of parathyroid disease during pregnancy [9]. 99mTc-MIBI planar imaging does not allow precise anatomical localisation and there is a large discrepancy with the actual position. Sestamibi scintigraphy combined with sestamibi SPECT has the highest positive predictive value and is the imaging method of choice for preoperative localisation in non-pregnancy. This test provides more precise preoperative localisation of parathyroid tissue and also greatly improves the accuracy of ectopic parathyroid tissue localisation [10].

There is no consensus on the treatment of PHPT in pregnancy and management needs to be tailored to the individual. Patients with asymptomatic and mild hypercalcaemia (serum-corrected calcium ≤2.85 mmol/L) can be treated conservatively during pregnancy with definitive surgical treatment after delivery, whereas in patients with symptomatic or moderate to severe hypercalcaemia (serum-corrected calcium ≥3.00 mmol/L), parathyroidectomy is the gold standard of treatment [11]. Foetal organ development is incomplete in early pregnancy and general anaesthesia in late pregnancy increases the risk of preterm delivery; surgery should be performed in mid-gestation, but in case of life-threatening complications in the mother, there is no need to take into account the gestational period and surgery should be performed immediately. ioPTH measurement is recommended to confirm the successful resection of hyperfunctioning parathyroid adenomas, and it is valuable in pregnant women with uncertain preoperative localisation of abnormal parathyroid tissue or with polyglandular disease [12].

Our patient was in severe acute pancreatitis with a serious condition, and considering the fact that the patient was in late pregnancy, bisphosphonates, and calcitonin may cause foetal abnormality [13], therefore, along with symptomatic support in the ICU, only conservative treatments such as simple rehydration, diuresis, and vitamin D supplementation were taken for PHPT. Afterwards, because of further aggravation of the patient’s condition, this forced us to abandon foetal preservation and perform a caesarean section. After delivery, the blood calcium level decreased, but was still higher than normal. After the pancreatitis was cured, the family considered that the patient’s body was weak, and she was discharged from the hospital for a period of time to recuperate before undergoing surgery. One month later, the patient was readmitted to the hospital for SPECT to clarify the location of the parathyroid adenoma, and was operated on with parathyroidectomy, in which the ioPTH was reduced to within the normal range, and the parathyroid adenoma was successfully removed. The patient was followed up by telephone one year after the operation, and he reported that he did not feel any discomfort.

## Conclusions

In conclusion, PHPT in pregnancy combined with severe acute pancreatitis is an extremely rare condition, and early diagnosis and treatment are essential to prevent complications such as severe vomiting and pancreatitis in pregnancy. Mild hypercalcaemia can be treated conservatively, but moderate to severe or symptomatic hypercalcaemia should be treated surgically. When the benefits of surgery outweigh the risks, especially in the second trimester, prompt surgical treatment is warranted.

## Data Availability

Not applicable.
